# Temporal dynamics of task switching and abstract-concept learning in pigeons

**DOI:** 10.3389/fpsyg.2015.01334

**Published:** 2015-09-02

**Authors:** Thomas A. Daniel, Robert G. Cook, Jeffrey S. Katz

**Affiliations:** ^1^Comparative Cognition Laboratory, Department of Psychology, Auburn UniversityAuburn, AL, USA; ^2^Avian Cognition Laboratory, Department of Psychology, Tufts University, MedfordMA, USA

**Keywords:** matching, non-matching, behavioral flexibility, concept learning, relational rule, reversal, pigeon

## Abstract

The current study examined whether pigeons could learn to use abstract concepts as the basis for conditionally switching behavior as a function of time. Using a mid-session reversal task, experienced pigeons were trained to switch from matching-to-sample (MTS) to non-matching-to-sample (NMTS) conditional discriminations within a session. One group had prior training with MTS, while the other had prior training with NMTS. Over training, stimulus set size was progressively doubled from 3 to 6 to 12 stimuli to promote abstract concept development. Prior experience had an effect on the initial learning at each of the set sizes but by the end of training there were no group differences, as both groups showed similar within-session linear matching functions. After acquiring the 12-item set, abstract-concept learning was tested by placing novel stimuli at the beginning and end of a test session. Prior matching and non-matching experience affected transfer behavior. The matching experienced group transferred to novel stimuli in both the matching and non-matching portion of the sessions using a matching rule. The non-matching experienced group transferred to novel stimuli in both portions of the session using a non-matching rule. The representations used as the basis for mid-session reversal of the conditional discrimination behaviors and subsequent transfer behavior appears to have different temporal sources. The implications for the flexibility and organization of complex behaviors are considered.

## Introduction

For any goal-directed behavior, an animal must selectively attend to the relevant cues in an environment while simultaneously ignoring irrelevant cues. An animal’s adaptability to these cues is known as behavioral flexibility (Aston-Jones et al., 1999; Shettleworth, 2009). An animal with high behavioral flexibility can readily switch between different relevant cues based on changes in the environment. Behavioral flexibility has been correlated with intelligence, and species that display high behavioral flexibility are on average considered to be more intelligent than those with low behavioral flexibility (Bitterman, 1965; Reader and Laland, 2002; Lefebvre et al., 2004; Roth and Dicke, 2005; Shettleworth, 2009; Reader et al., 2011).

The mid-session reversal procedure requires such behavioral flexibility because the relevance of available cues in the task dynamically changes with time, as the reinforcement contingencies are reversed in the middle of a session. For example, selecting the green icon in a simultaneous task is reinforced for the first half of a session, and then halfway through the session selecting the red icon is reinforced. This mid-session reversal thus requires the subject to adapt its behaviors flexibly within a session to respond optimally.

Cook and Rosen (2010) found that pigeons did not behave optimally in a mid-session reversal task. In their two-alternative conditional choice task, the first half of a session was a matching-to-sample (MTS) task and the second half of the session used a non-matching-to-sample (NMTS) task. Both tasks used the same red and cyan circles as stimuli. In the MTS task, for example, the pigeon was presented with a sample stimulus (e.g., red circle), completed an observing response, and then was presented with two choice stimuli (e.g., red circle and cyan circle) that were equidistant to the left and right of the sample. The correct response was to choose the comparison stimulus that matched the sample. NMTS was identical to MTS, except the correct response was to choose the comparison stimulus that did not match the sample. Thus, during the first half of a session the pigeons had to learn “if cyan circle then peck cyan circle” and in the second half of a session the pigeons had to learn “if cyan circle then peck red circle” (and with corresponding rules for red samples). Hence, to perform well, pigeons had to learn to switch at the midpoint of a session MTS to NMTS behaviors. If pigeons performed optimally they should have learned to respond exclusively based on matching rules during the first half of the session then switched to non-matching rules at the midpoint of the session. Pigeons did not respond optimally. Cook and Rosen (2010) found that before the reversal, pigeons began to anticipate the NMTS contingency and switched to pecking the incorrect comparison prematurely. Likewise, after the reversal, the pigeons perseverated on the formerly correct comparison for too long. This effect has been replicated in a number of subsequent studies testing simpler discriminations and other species (Rayburn-Reeves et al., 2013a,b; McMillan et al., 2014, 2015; McMillan and Roberts, 2015). According to Cook and Rosen (2010), these errors occurred because pigeon behavior was controlled less by the outcome of the previous trial than by internal temporal factors (i.e., time). The anticipatory and perseverative errors occurred on a learned time course, with switching behavior mediated by temporal cues rather than the quantity of trials or the outcomes of previous trials. This temporal control over responding has been confirmed by studies also manipulating time-related factors such as the inter-trial interval (McMillan and Roberts, 2012; Rayburn-Reeves et al., 2013a) or when the reversal points vary from session to session rather than remain fixed (Rayburn-Reeves et al., 2013b).

In the present study we were interested in testing whether temporal factors would similarly control switching between relational rules in a mid-session reversal task. In all previous studies, only Cook and Rosen (2010) involved conditional discriminations where relational rules could have been learned. Given that the training set of stimuli involved only two items, it is likely pigeons learned via item-specific rules. It is well established that such small training sets generally result in item-specific learning (Katz et al., 2007). To generate relational learning, so that the relationship between the items is learned (i.e., abstract-concept learning: “peck the matching stimulus” or “peck the non-matching stimulus”), it is best to use the largest training sets possible. To promote the formation of relational rules in the present task, we took advantage of two factors. The first is that we tested the animals with larger set sizes that have previously supported relational transfer. Second we used two groups of pigeons that had previously demonstrated full-concept learning in either MTS or NMTS using such larger training sets. We will refer to these two groups as the matching-concept group (MCG) and non-matching-concept group(NMCG) based on this prior experience. The MCG was trained in MTS and eventually demonstrated full concept learning (Bodily et al., 2008). They were initially trained with a small set size of three stimuli and showed no transfer to novel stimuli. These pigeons then had their training set systematically doubled eight times to a final set size of 768 stimuli. After reaching a performance criterion at each set size they were tested with trial-unique novel stimuli. Transfer performance increased at the smallest set size from chance (50%) to equivalent to baseline and over 80% by then end of set-size expansion. Such transfer constitutes full abstract-concept learning (Katz et al., 2007). The NMCG was trained exactly like the MCG including the same stimuli, sessions, and apparatus. The only procedural difference between the groups was that the NMCG was rewarded for pecking the non-matching, different comparison stimulus, whereas the MCG was rewarded for pecking the matching comparison stimulus. Similar to the MCG, the NMCG demonstrated full concept learning by the end of set-size expansion (Daniel et al., 2015).

These two groups of pigeons served as subjects in the present experiment. The first half of each session used a MTS task, and the second half used an NMTS task. Implementing a set-size expansion procedure, the initial training set size consisted of three stimuli, and this was progressively doubled to 6, then 12 total stimuli. After acquiring the mid-session reversal contingency with the 12-item set, abstract-concept learning was tested by placing novel stimuli at the beginning and end of test sessions. Several questions were of interest: first, previous mid-session reversals used 2 or 3 stimuli, and the effect of additional stimuli (i.e., 6 or 12) is still unknown. Cook and Rosen (2010) attempted to train a three-alternative conditional discrimination, but found that learning all three possible outcomes to each sample was very difficult within the same sessions (eventually they learn two). Second, the impact of prior concept learning (i.e., MTS or NMTS) has yet to be tested in mid-session reversals. We anticipated that the matching and NMCGs would show an early advantage in their respective MTS- and NMTS- half of the mid-session reversal, but that the advantage would disappear over training. Third, it is unclear to what level pigeons could learn the mid-session reversal task with the increasing number of competing contingencies. That is, if the pigeons learned item-specific rules, then the number of competing contingencies increases as the set size expands. Finally, it is unknown if pigeons can apply relational learning in a mid-session reversal, particularly with the expanded set size. We anticipated only partial concept learning given previous results with set sizes of 12 stimuli (Katz et al., 2010). Nonetheless, such conditions, along with the birds’ previous experience, should be sufficient to see if pigeons could show flexibly switching using matching and non-matching-concepts within the same session.

## Materials and Methods

### Subjects

Six male pigeons (*Columba livia*) from the Palmetto Pigeon Plant served as subjects. Subjects were maintained within 80–85% of their free-feeding body weight throughout the study; in the event that a subject’s weight fell above or below this range for the day, it did not participate in that day’s session. Subjects resided in a colony room governed by a 12 h light/dark cycle and were housed individually with free water and grit access. All subjects previously demonstrated full transfer in MTS (Bodily et al., 2008) or in NMTS (Daniel et al., 2015). The three subjects that learned the *matching* concept will hereby be referred to as the MCG, and the remaining three subjects that learned the *non-matching-concept* will be referred to as the NMCG.

### Apparatus

Pigeons were tested using custom wood (35.9-cm wide × 45.7-cm deep × 51.4-cm high) test chambers. A fan (Dayton 5C115A, Niles, IL, USA) located in the back wall of each chamber provided ventilation and white noise. The computer detected pecks via an infrared touch screen (17” Unitouch, Carroll Touch, Round Rock, TX, USA). This pressure-fit touch screen sat within a 40.6-cm × 32.1-cm cutout in the front panel that was centered 7.7 cm from the top of an operant chamber. A 28-V (No. 1829, Chicago Miniature, Hackensack, NJ, USA) houselight, located in the center of the ceiling, illuminated the chamber during intertrial intervals (ITI). A custom hopper containing mixed grain could be accessed through an opening (5.1 cm × 5.7 cm) centered in the front panel 3.8-cm above the chamber floor.

Custom software written with Visual Basic 6.0 on a Dell Optiplex GX110 recorded and controlled all events in the operant chamber. A video card controlled graphics generated by the computer while a computer-controlled relay interface (Model no. PI0-12, Metrabyte, Taunton, MA, USA) maintained operation of the grain hopper and the lights to both the hopper and the chamber.

### Stimuli

Visual stimuli were computer-created, color cartoon JPEG images that were 2.5-cm high × 3-cm wide at 28 pixel/cm (cf. Katz et al., 2008, **Figure 2**). All stimuli used were of similar size and shape but were distinguishable from one another. Each sample stimulus and comparison stimulus appeared at approximately 8 cm above the bottom of the monitor directly above the grain hopper. The center of the left and right comparison stimuli appeared 8.5 cm from the center of the sample.

### Training

Pigeons were initially trained with a set size of three stimuli (apple, duck, and grape). Trials began with a round white circle displayed on the monitor (in the same position as the sample) as a ready signal. Once pigeons pecked the white circle once, the sample stimulus appeared. Pigeons pecked the sample 20–25 times (randomly selected); this pecking requirement began with one peck but was systematically increased over approximately eight sessions to 20–25 pecks. After pigeons completed the response requirement, two comparison stimuli were presented: one comparison stimulus matched the sample and the other did not. Daily sessions were conducted 5–7 days a week, with each session comprised of 96 trials. In the first half of the session (i.e., trials 1–48), a response to the matching comparison resulted in grain reinforcement. In the second half of the session (i.e., trials 49–96), a response to the non-matching comparison resulted in grain reinforcement. Grain access was between 2 and 3.5 s of mixed grain depending on the pigeon’s body weight prior to the session. An incorrect choice response resulted in an unlit 5-s timeout. All trials were followed by a 3-s ITI whether the response was correct or incorrect. With a set size of three stimuli, there were 12 possible combinations. Each combination appeared 8 times per 96-trial session. Stimuli were pseudorandomized to ensure that a combination would not directly repeat on the next trial. Correct response locations (left or right) were counterbalanced so that an equal number of correct left and right responses occurred in any given session.

A correction procedure required subjects to repeat any incorrect trials until a correct response was made, but only the first response to each trial was counted and computed for accuracy. The correction procedure was used at the onset of training and for maintenance when a side-bias developed toward left or right comparisons. Training continued until a pigeon reached above a mean 65% accuracy during the first and last eight trials of a session across 10 consecutive sessions without correction procedure, or until they experienced at least 100 sessions of training. Only sessions without correction procedure were analyzed. This performance-based criterion was created to ensure that pigeons were reliably matching and non-matching above chance before advancing to larger training sets.

### Set-Size Expansion

Once the performance-based criterion was reached, an equal number of new training stimuli were added to the previous training set. The number of images used in training increased from 3 to 6, and then to 12. Each training set retained the stimuli from the previous training set. For each session, sample and comparison stimuli were pseudorandomly assigned from the stimulus set. Each session consisted of 96 trials counterbalanced for left/right-correct. After pigeons reached criterial performance at the 12-item set size transfer testing began on the next session.

### Transfer Testing

Transfer testing was comprised of eight consecutive sessions without correction procedure. Each testing session contained 96 trials (88 baseline and 8 transfer trials). Within each testing session, two transfer trials occurred within four blocks of eight-trial blocks (Trials 1–8, 9–16, 81–88, 89–96). The first and last two blocks of a session were used to capture the highest level of matching and non-matching performance in the task. Left and right correct responses were counterbalanced within each eight-trial block. All transfer trials were novel, so each cartoon image was only used once during transfer testing. Hence, with each configuration composed of two different images, there were 16 novel cartoon images in each testing session for a total of 128 (16 stimuli × 8 sessions) novel cartoon images. Responses on transfer trials were reinforced identically to baseline trials. A correction procedure was not used at any point during transfer testing. Baseline trials were also counterbalanced for left and right responses using the same specifications as those during training.

## Results

### Acquisition

**FIGURE 1:**
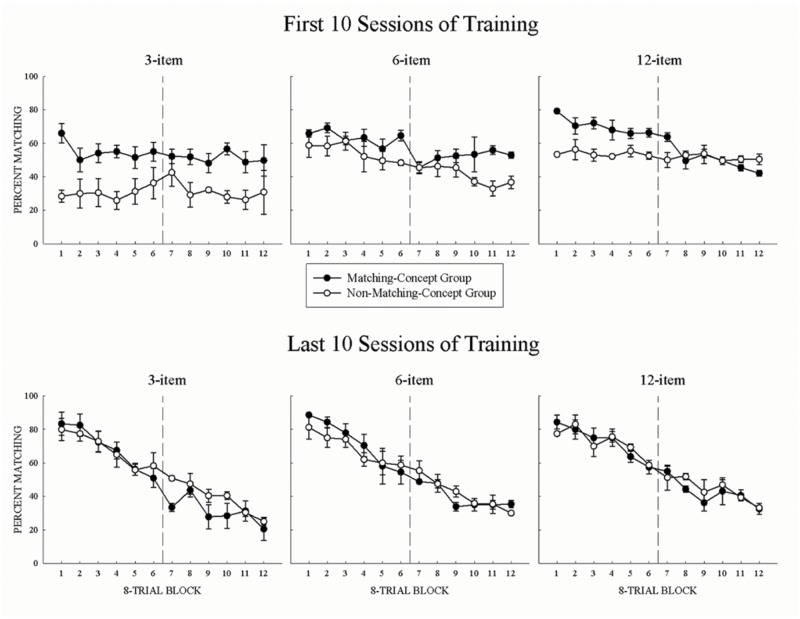
**(Top panels)** Mean percent correct matching for the first 10 sessions of acquisition across 8-Trial Blocks for the matching-concept group (MCG; filled symbols) and non-matching-concept group (NMCG; open symbols). **(Bottom panels)** Mean percent correct matching for the final 10 sessions of acquisition across 8-Trial Blocks plotted by group. Error bars represent SEMs.

The first 10 sessions of acquisition for all set-sizes are shown in the top panels of **Figure 1**. In **Figure 1**, the percentage matching choices are plotted for the matching-concept (filled circles), and non-matching-concept (open circles) group. High values indicate matching behavior while low values indicate non-matching behavior. For all set-sizes, both groups’ initial choice behavior was mostly stable across a session. This result shows the pigeons were not strongly task-switching as a function of the mid-session reversal. Instead, early acquisition was often characterized by pigeons making many choice errors across the session with little or no savings from training with the previous set-sizes. Separate two-way repeated-measure ANOVA of Group (matching, non-matching) × 8-Trial Block (1 – 12) for each set size revealed main effects of Group, *F*s(1,2) > 27.2, *p*s < 0.05, $\eta_{p}^{2}$s > 0.96. This suggests that prior conceptual training of each group had a significant effect on behavior at the start of each set-size acquisition, with the MCG tending to match and the NMCG tending to non-match. These group differences may have emerged because the NMCG had a shorter break in testing between the end of the prior experiment and the start of the present experiment than the MCG (>1 year). Also, the group differences may have emerged because MCG had responses to their experience-congruent concept reinforced for the first portion of the session, while NMCG had to make experience-incongruent concept responses in the first half of the session and then switch to experience-congruent responses in the second portion. If we had tested non-matching in the first portion and matching in the second half of the session the acquisition results may have been different. Main effects of 8-Trial Block were found only for the 6-item and 12-item training sets, *F*s(11,22) > 12.8, *p*s < 0.01, $\eta_{p}^{2}$s > 0.87, indicating that pigeons were starting to engage in task-switching even within the first 10 sessions of acquisition as they become more familiar with the task. An interaction between the two factors was found only at the 12-item set, *F*(11,22) = 3.1, *p* < 0.05, $\eta_{p}^{2}$ = 0.61, due to the MCG engaging in task-switching, while the NMCG did not. This was confirmed by examining the slope of the percent matching function across a session, with only the MCG significantly different from 0 (MCG: *M* = -3.2; NMCG: *M* = -1.76).

By the last 10 sessions of acquisition, all set-sizes supported good mid-session reversal for each group. This is shown in the bottom three panels of **Figure 1**. Pigeons were able to learn to conditionally match or non-match across a session (3-item = 41 sessions, 6-item = 52 sessions, 12-item = 58 sessions). One pigeon from each group failed to reach criterial performance at the 12-item training set. Thus, unlike the top panels, the bottom panels show a functional transition between matching and non-matching behavior, and that pigeons were engaged in both the anticipatory and perseverative errors common in mid-session reversal (cf. Cook and Rosen, 2010). For all set-sizes, pigeons began the session matching and then linearly reverse responding to non-matching, as indicated by the negative linear functions for both groups at all set sizes. Separate two-way repeated-measure ANOVA of Group (matching, non-matching) × 8-Trial Block (1 – 12) for each set size found no main effects of Group, but did reveal main effects of 8-Trial Block for all set-sizes *F*s(11,22) > 26.2, *p*s < 0.01, $\eta_{p}^{2}$s > 0.96. Subsequent trend analyses show that percent matching decreased linearly across the session, confirming that the pigeons engaged in task-switching behaviors across all set-sizes, *F*s(1,5) > 210.2, *p*s < 0.01, $\eta_{p}^{2}$s > 0.99. Thus, prior learning (i.e., MCG or NMCG) did not have a significant impact on their terminal reversal behavior once the pigeons reached criterial performance.

### Transfer

Over transfer testing, all pigeons maintained the linear decrease of matching performance within a session found during task switching. The pigeons readily transferred to novel stimuli. In regard to relational learning, the MCG applied a matching relational rule when presented with novel stimuli during the matching *and* non-matching halves of the task switching procedure. In contrast, the NMCG applied a non-matching relational rule when presented with novel stimuli during the non-matching *and* matching halves of the task switching procedure.

**FIGURE 2:**
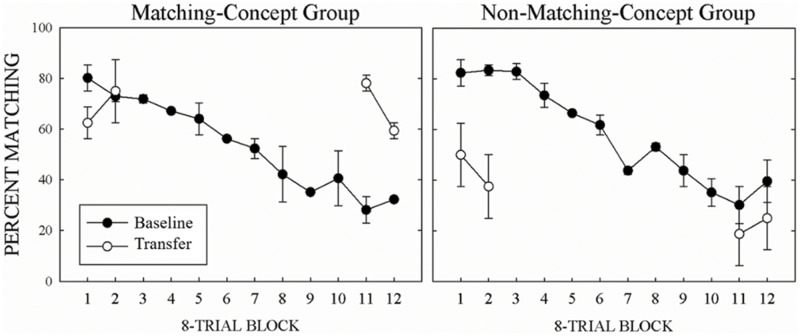
**Mean percent correct matching for baseline (filled symbols) and transfer (open symbols) with the MCG in the left panel and the NMCG in the right panel.** Error bars represent SEMs.

**Figure 2** shows mean percent matching across 8-Trial Blocks for baseline (filled circles) and transfer (unfilled circles) trials for the MCG on the left and the NMCG on the right. With trained stimuli, both groups (NMCG: *M* = -5.2, MCG: *M* = -4.8) continued to show a decrease in percent matching across Trial Blocks. The MCG transferred to novel trials in the first two Trial Blocks when required to match, but when required to non-match for the last two Trial Blocks they did not. The opposite pattern of behavior was shown for the NMCG. That is, the NMCG transferred to novel trials in the last two Trial Blocks when required to non-match, but when required to match for the first two Trial Blocks they did not. These results were confirmed by a three-way interaction, *F*(3,3) = 16.2, *p* < 0.05, $\eta_{p}^{2}$ = 0.94, from a three-way repeated-measures ANOVA of Group (matching, non-matching) × Trial-Type (baseline, transfer) × 8-Trial Block (first, second, eleventh, twelfth) on percent matching. To analyze each pigeon’s transfer during matching, we compared the 32 transfer trials to the mean accuracy of the baseline trials from the first two and last two 8-Trial Blocks from the eight transfer sessions using one-sample *t*-tests. At the first two 8-Trial Blocks, for the two matching-concept pigeons, transfer was equal to baseline (L822, *p* = 1, S8288, *p* = 0.09) and for the two non-matching-concept pigeons, transfer was lower than baseline [D7, *t*(31) = 3.4, *p* < 0.01; L5, *t*(31) = 5.8, *p* < 0.01]. At the last two 8-Trial Blocks, for the two matching-concept pigeons, transfer was higher than baseline [L822, *t*(31) = 3.5, *p* < 0.01; S8288 *t*(31) = 5.4, *p* < 0.01] and for the two non-matching-concept pigeons, transfer was equivalent to baseline for one pigeon (D7, *p* = 1) and lower than baseline for one pigeon [L5, *t*(31) = 5, *p* < 0.01].

## Discussion

The present experiment shows that pigeons can learn a mid-session reversal involving conditional discriminations with set sizes up to 12 stimuli. With trained stimuli, MTS and NMTS discrimination was bound to the temporal cues of each session, replicating the main finding from Cook and Rosen (2010) showing a modulation of task switching over a session. The pigeons made errors of anticipation before the reversal and errors of perseveration after the reversal at all set sizes. Unlike with normal expansion of set sizes, however, the pigeons show little savings across successive acquisitions of these tasks with the added stimuli. Thus, based on acquisition data alone, the pigeons showed no evidence of relational rule use within each MTS and NMTS portion of a session. When tested for relational rule use with novel stimuli, pigeons reverted back in all tests to their prior MTS or NMTS rules learned prior to the mid-session reversal training. Thus, the MCG applied the abstract matching rule to all novel stimuli, regardless of when the novel stimuli were presented in the session. The NMCG applied the abstract non-matching rule to all novel stimuli. These results suggest differences in the temporal dynamics of how trained and untrained stimuli are processed by the pigeons. One effect of training is that it binds familiar stimuli to the different and competing MTS and NMTS behaviors temporally required across a session. Novel stimuli, in contrast, are not bound to time in this way and as a result, the pigeons use their previous learned rules to respond to them.

As a consequence, it appears the pigeons learned the present conditional discrimination mid-session reversal task by learning item-specific rules that were bound to the session’s time-course. For example, pigeons learned rules such as “if grape is the sample, then peck the grape in the first half of the session,” and “if grape is the sample, then peck the apple or duck in the second half of the session.” This learning can be contrasted with if the pigeons had learned separate abstract concepts bound to the first and second half of a session. Such relational rules would have been “if it is the first half of the session, peck the matching picture” and “if it is the second half of the session, peck the non-matching picture.”

This focus on item-specific, rather than conceptual, learning may have stemmed from the need to deal with the competing behaviors required to the shared stimuli of each portion of the session. It certainly would explain why adding more exemplars to the task did not benefit learning as established previously (Bodily et al., 2008; Daniel et al., 2015). Every expansion of the set size presented a new challenge and apparently a need for new learning by the birds. This issue was in part responsible for why we tested concept learning with novel stimuli after finishing our expansion to a set size of 12. It was not clear to us that a 24-item set size would be manageable, at least not without extensive additional training. Thus, the pigeons reacted differently to set-size expansion than that found in the previous successful concept studies. Using the same three stimuli (duck, apple, grape) pigeons have acquired MTS and NMTS in less than 11 sessions (Bodily et al., 2008; Daniel et al., 2015), a rate fourfold less than the pigeons in the present experiment. In addition, when the set size was expanded to 6 and 12 stimuli acquisition decreased relative to the initial acquisition with three stimuli in both MTS and NMTS. In contrast, in mid-session reversal learning acquisition increased with expansion further indicating item-specific learning.

The difficulty of this item-specific learning may also be responsible for the highly linear switching function seen in this experiment. Switching between the matching and non-matching discrimination within the same session was always quite gradual for the pigeons. Previous switching functions found in mid-session reversal with simpler discrimination contingences are typically not linear, with extended periods of good performance at the beginning and end of sessions. Perhaps because the many individual items, were spread out over the whole session, binding them to specific portions of the session was difficult, resulting in the observed linear switching function. If rule-based concepts had been learned, a more marked sigmoidal switching function would have been strongly expected.

The absence of concept learning across the mid-session reversal task was further evidenced by the lack of novel stimulus transfer across a session. Instead of conditional transfer depending on temporal location, the groups performed quite differently during transfer. Both groups reverted back to their prior matching and non-matching-concept learning experience to discriminate these novel stimuli. The MCG applied the matching concept to all novel stimuli, regardless of whether it was presented before or after the mid-session reversal point. In an identical manner, the NMCG applied the non-matching-concept to all novel stimuli. Thus, when untrained stimuli were introduced, they were unbound from the session’s time course needed to support item-specific learning. Without such temporal cues, the pigeons then relied on their previous matching and non-matching-concept learning experience to solve the trial.

The latter reversion back to previously learned rules may best be explained within a framework similar to behavioral renewal (Bouton, 2002, 2004). Behavioral renewal occurs when an animal learns a behavior in one context, is given a second context where that original behavior is extinguished, followed by a return to the first context. If, after extinction, the animal behaves in accord with the first context, the previous behavior is “renewed” (Bouton, 2002). In our experiment, both groups were trained in an abstract-concept learning task, serving as the first context. Then, these pigeons were trained in a mid-session reversal task, serving as the second context. When novel stimuli appeared in transfer trials, it created an ambiguous context since they had no reversal history to bind them to time within a session, and thus pigeons relied on the previous learning of their first context (i.e., abstract-concept learning). Because no item-specific rules had yet been formed for these new stimuli, pigeons responded to these trials like transfer trials from their first context and applied the abstract relational matching or non-matching-concept.

In the future, it will be of interest to see how naïve pigeons trained with large set sizes that have not learned a prior abstract matching or non-matching-concept discriminate novel stimuli across the different portions of a mid-session reversal. Such naïve pigeons would not have a prior behavior to “renew” when given an ambiguous context (i.e., a transfer trial). They may fail to transfer completely. This would suggest that competing relational rules (matching and non-matching) may be very hard to learn within the same session. Alternatively, they may transfer based on the configuration’s placement in the session. If so, it would suggest that abstract concepts can be differentially bound to the context of the within session time course.

In summary, these results add to the literature demonstrating the behavioral and cognitive flexibility of pigeons (Cook, 2001; Shettleworth, 2009; Cook and Rosen, 2010; Qadri and Cook, 2015). Our results indicate that the dynamics in pigeons for a mid-session reversal task are bound to the temporal time course within a daily session for trained stimuli. When stimuli have no prior association with the temporal dynamics within the mid-session reversal task, pigeons rely on an abstract concept that is unbound from time and perhaps had temporal priority due to renewal-like processes. These results warrant further investigation: what impact does previous experience have on transfer? Would this renewal effect remain using a different procedure or multiple reversal points (McMillan et al., 2015)? More information is needed to understand these mechanisms, and pigeons serve as an excellent model to understand the comparative processes that underlie the temporal dynamics of discrimination learning and concept formation.

## Ethical Standards

This experiment complied with current United States law and following the relevant ethical guidelines for animal research (IACUC approved and conducted in AAALAC approved facilities).

## Conflict of Interest Statement

The authors declare that the research was conducted in the absence of any commercial or financial relationships that could be construed as a potential conflict of interest.
